# Drug Delivery by Tattooing to Treat Cutaneous Leishmaniasis

**DOI:** 10.1038/srep04156

**Published:** 2014-02-24

**Authors:** Marina Temi Shio, Marilene Paquet, Caroline Martel, Tom Bosschaerts, Stef Stienstra, Martin Olivier, Anny Fortin

**Affiliations:** 1 Departments of Medicine and of Microbiology and Immunology, The Research Institute of the McGill University Health Centre, Faculty of Medicine, McGill University, 3775 University Street, Montreal, Canada; 2 Department of Microbiology, Immunology and Parasitology, Universidade Federal de São Paulo, R. Botucatu 862, São Paulo, Brasil; 3 Département de pathologie et de microbiologie, Faculté de Médecine Vétérinaire, Université de Montréal, Sicotte 3200, St-Hyacinthe, Canada; 4 Dafra Pharma Research & Development, Slachthuisstraat 30, Turnhout, Belgium; 5 MT-Derm, Gustav-Krone-Straβe 3, Berlin, Germany; 6 Department of Biochemistry, McGill University, 3655 Promenade Sir-William Osler, Montreal, Canada

## Abstract

This study establishes a proof-of-concept that a tattoo device can target intra-dermal drug delivery against cutaneous leishmaniasis (CL). The selected drug is oleylphosphocholine (OlPC) formulated as liposomes, particles known to be prone to macrophage ingestion. We first show that treatment of cultured *Leishmania*-infected macrophages with OlPC-liposomes results in a direct dose-dependent killing of intracellular parasites. Based on this, *in vivo* efficacy is demonstrated using a 10 day tattooing-mediated treatment in mice infected with *L. major* and *L. mexicana*. In both models this regimen results in rapid clinical recovery with complete regression of skin lesions by Day 28. Parasite counts and histopathology examination confirm high treatment efficacy at the parasitic level. Low amount of drug required for tattooing combined with fast clinical recovery may have a positive impact on CL patient management. This first example of tattoo-mediated drug delivery could open to new therapeutic interventions in the treatment of skin diseases.

Leishmaniasis is a vector-borne disease that is caused by obligate intra-macrophage protozoa of the *Leishmania* species. Leishmaniasis can cause different clinical syndromes, including cutaneous leishmaniasis (CL), in which the patient generally presents with one or several ulcer(s) or nodule(s) on the skin, resulting from the infection of phagocytic cells located in the dermis. Most of the twelve million people infected with *Leishmania* worldwide are CL cases, an 1.5 million new cases occur annually^1^.

Although there is a huge clinical need, no good treatment is available for CL at the moment. Intra-lesional injections of anti-parasitic pentavalent antimonials such as stibogluconate or meglumine antimoniate are currently the gold standard treatment in several endemic countries, but these treatments present the disadvantages of being long, painful and distressing, especially for children presenting facial lesions. In addition these injections are not recommended in case of lymphatic invasion and peri-cartilaginous (ears, nose), peri-articular or peri-orificial lesion topographies. Systemic treatments with antimonials, pentamidine, amphotericin B or oral miltefosine represent valuable alternative options, but their unwanted side-effects often lead to unfavorable risk-benefit ratios for CL patients, translating into non-recommendation for treatment in several cases^1^.

An alternative approach to the dermatological problem of CL is a topical formulation such as cream and ointment. The advantages of such treatment are better compliance by the patients, reduced costs and avoidance of systemic toxicity. However, development of topical anti-leishmanial drugs necessitates a good absorption and retention of the active ingredient at the site of infection in the dermis, and this can be difficult depending on the chemical class of drugs and the type (or stage) of lesions to be treated^2^.

This study investigates the use of the tattooing technology as a new delivery system to target an anti-leishmanial drug to the dermis. The tattooing procedure is robust, easy to use, fully hygienic and beneficiates from decades of development by the cosmetic industry. Tattooing administration has already been used in medical research to define a DNA vaccination strategy^3^. However, to our knowledge the current study is the first to investigate drug-delivery via tattooing to treat a skin disease. The selected drug for this proof-of-concept project is oleylphosphocholine (OlPC), an alkylphosphocholine formulated as liposomes of about 100 nm which can be directly used for tattooing. In a previous study, the OlPC active pharmaceutical ingredient showed good *in vitro* activity against several CL-causing strains of *Leishmania* (*L.*), including *L. major*, *L. tropica*, *L. mexicana, L. panamensis* and *L. braziliensis*^4^. These data prompted us to study the *in vivo* efficacy of this drug against CL when formulated as liposomes and delivered with a tattoo instrument while comparing to other, more classical, administration routes.

## Results

### In vitro activity of oleylphosphocholine (OlPC) against CL parasites

**Figure 1:**
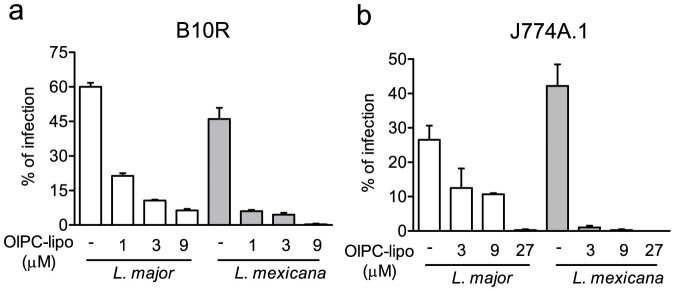
OlPC-liposomes induce dose-dependent killing of intracellular *Leishmania* in cultured macrophage-derived cell lines. Macrophage-derived cell lines were infected with ratios of 1 cell:20 parasites (B10R; a) or 1 cell:10 parasites (J774A.1;b) of *L. major* or *L. mexicana* for 6 h and treated with indicated concentration of OlPC-liposomes (OlPC-lipo) for 18 additional hours. Data are shown as mean ± SEM of percentage of *Leishmania*-infected macrophages (3–5 counts of 100 cells from a representative experiment out of 2 different experiments).

The initial part of this work aimed to establish the potency of OlPC-liposomes to kill the extracellular (promastigote) and intracellular (amastigote) forms of *Leishmania* parasites using *in vitro* models. OlPC-liposomes were first tested against *Leishmania* promastigotes; cultures of different *Leishmania* strains in log phase of growth were exposed to increasing concentrations of the drug for 24 hrs, after which the parasite concentration was measured. OlPC-liposomes induced dose-dependent killing of promastigotes, with inhibitory concentrations (IC50) ranging between 1.1 and 8.0 μM (Table 1). Subsequently, B10R and J774.1 macrophage-derived cell lines infected with *L. major* or *L. mexicana* amastigotes for 6 hours were exposed to three increasing concentrations of OlPC-liposomes for 18 hrs. Drug exposure resulted in a dose-dependent reduction of parasitized cells as evaluated by light microscopy (Figure 1). For instance, exposure to 9 μM of liposomal OlPC led to a 60 to 90% reduction of the proportion of *L. major* infected cells compared to untreated, and to a 99% reduction for *L. mexicana* infected cells. This data supports previous observations made with an aqueous formulation of OlPC in infected primary peritoneal mouse macrophages^4^. Treatment of macrophage-derived cells (non-infected and infected) with OlPC-liposomes did not induce ROS and/or NO production above control levels, supporting the fact that the drug-mediated cidal effect on intracellular amastigotes does not require macrophage-mediated microbicidal mechanisms (see Supplementary Fig. 1).

### Tattoo-mediated treatment of CL infected mice

**Figure 2:**
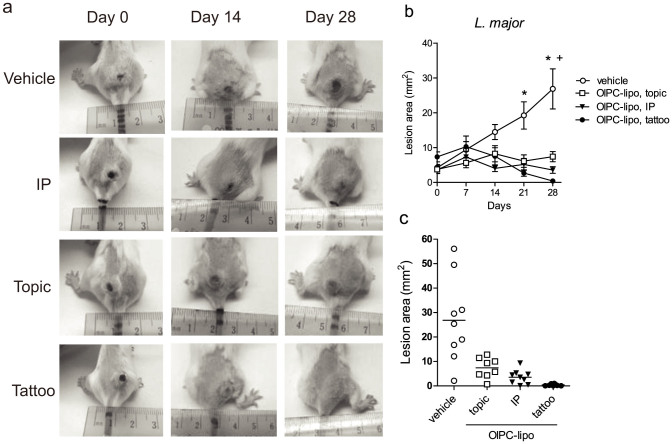
*In vivo* efficacy of OlPC-liposomes against *L. major* CL. Female Balb/c mice received 10 × 10^6^ stationary phase *L. major* promastigotes transfected with luciferase at the tail base. After 12 weeks, mice were treated 2 × 5 days with PBS vehicle or OlPC-liposomes: 36–90 μg/day administered with a tattoo device (tattoo), 30 mg/kg/day IP, or 900 μg/day as a topical drop on the top of the lesion (topic). Lesions were measured from the start day of the treatment (Day 0) on individual pictures taken on Day 0, 7, 14, 21 and 28 for each mouse. Lesion areas were calculated using the Adobe Photoshop® software. Representative pictures of lesions from each group are shown in (a); mean lesion sizes (±SEM) up to Day 28 are presented in (b); P < 0.05 between treated (*tattoo), (^+^IP) and vehicle groups (n = 7–9). Individual lesion sizes per group on Day 28 are shown in (c).

Skin tattooing of DNA for vaccination has previously shown to transfect cells distributed over the upper layers of the dermis and the epidermis^3^,^5^. Since CL parasites replicate in the upper layer of the dermis, the tattoo device was used to target the delivery of OlPC-liposomes in this region of the skin (Figure 2). Female Balb/c mice were infected at the tail base with stationary phase promastigotes transfected with luciferase. When lesions became visible (necrotic center of about ~5 mm^2^), mice were grouped and treated 2 × 5 days with OlPC-liposomes or PBS vehicle using the tattoo-device. Each tattooing session consisted of 12 two-second administrations with a 5-needle head oscillating at 100 Hz, for a total of 12000 punctures distributed equally around the lesion borders. We estimate that ~2–5 μl of OlPC-liposomes (36–90 μg) is injected into the skin during every tattooing session. Two other routes of administration were used to compare efficacy, also using a 2 × 5-day regimen: a systemic route with 30 mg/kg/day of OlPC-liposomes (about 600 μg of OlPC/day) given by intraperitoneal injections (IP), and topical route with 50 μl of OlPC-liposomes (900 μg of OlPC/day) directly dropped on top of the lesion (referred as “topic”). Lesion sizes were followed from the start day of the treatment (Day 0) up to Day 28, and pictures of individual mice were taken on Day 0, 7, 14, 21 and 28 (see representative pictures on Figure 2a).

**Figure 3:**
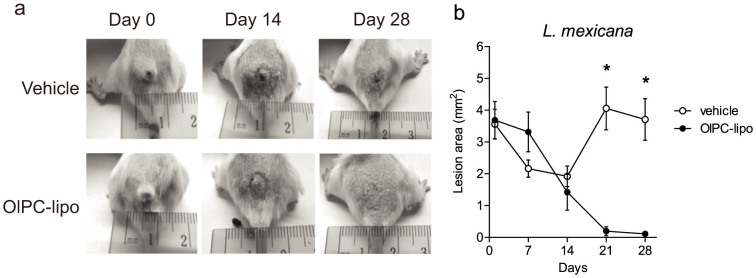
*In vivo* efficacy of OlPC-liposomes against *L. mexicana* CL. Female Balb/c mice received 10 × 10^6^ stationary phase *L. mexicana* promastigotes at the base of tail. After 12 weeks, mice were treated 2 × 5 days with PBS vehicle or OlPC-liposomes (36–90 μg/day) administered with a tattoo device. Lesions were measured from the start day of the treatment (Day 0) from individual pictures taken on Day 0, 7, 14, 21 and 28 for each mouse. Lesion areas were calculated using the Adobe Photoshop® software. Representative pictures of lesions from each group are shown in (a), while mean lesion sizes (±SEM) up to Day 28 are presented in (b); (*) P < 0.05 between treated and vehicle groups (n = 6).

A measurable effect of the drug was observed for all treatment routes tested (Figure 2b and c). Dropping the liposomes on top on the lesions impacted lesion sizes on Day 28 (7.4 mm^2^, SEM ± 1.5 compared to 26.9 mm^2^, SEM ± 5.8 in vehicle-treated), although statistically non-significant. In contrast, IP treatment with OlPC-liposomes had a significant impact on lesion size on Day 28 (mean lesion sizes of 3.6 mm^2^; SEM ± 1.0), translating a good efficacy of the drug when administered systemically. Importantly, the tattooing-mediated treatment led to a complete (or nearly complete) disappearance of the lesions after 10 days of treatment (mean size 0.4 mm^2^, SEM ± 0.1 on Day 28). The efficacy of tattooing-mediated treatment of CL lesions with OlPC-liposomes was reproduced independently in Balb/c female mice infected with *L. mexicana* promastigotes using the same protocol. In general, the *L. mexicana*-induced lesions grew slower than the *L. major*-lesions. In these mice, 10 days (2 × 5) of tattooing with OlPC-liposomes resulted in a mean lesion size of 0.1 mm^2^ (SEM ± 0.05) on Day 28 compared to 3.7 mm^2^ (SEM ± 0.7) in PBS-tattooed animals (Figure 3).

In general, all mice tolerated the drug treatments (all routes of administration tested) without any weight loss or other signs of toxicity. All experiments using the tattooing procedure left the mice with normal skin macroscopic appearance, and hair growth remained normal (not shown).

**Figure 4:**
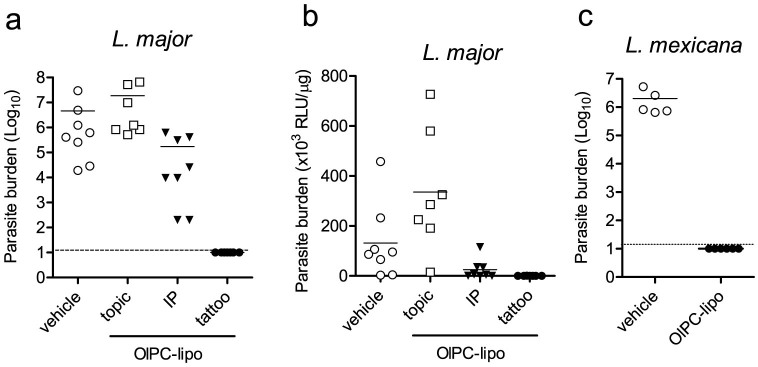
OlPC-liposomes reduce parasite burden in CL lesions induced by *L. major* and *L. mexicana*. On Day 28 following start of treatment, 5 to 8 mice from each treated groups were sacrificed and individual lesions were disinfected, excised and homogenized to perform (a and c) limiting dilution and/or (b) luciferase assay (mice infected with Friedlin-luciferase *L. major*). Dashed lines represent the limit of detection of the dilution assay.

To correlate clinical findings with parasitological data, mice from different treatment groups were sacrificed on Day 28 and lesions were excised, homogenized, and parasite quantification was performed by limiting dilution and/or luciferase assays (Figure 4). As expected from the clinical observations, the assays detected the presence of remaining parasites in the lesions of the topically treated animals and in those treated by IP injections. On the other hand, parasite levels in the animals treated by tattooing were below limits of detection in both assays.

**Figure 5:**
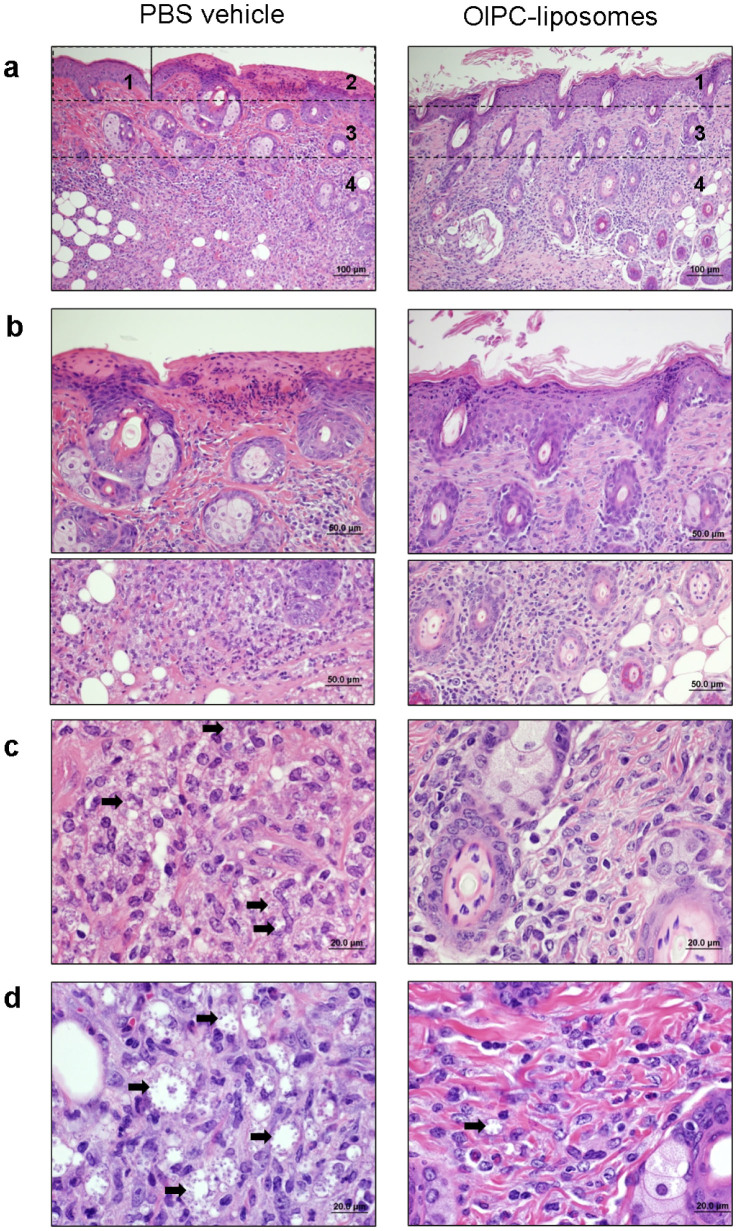
Tattoo-mediated administration of OlPC-liposomes reduces parasite burden and inflammation in the CL lesions induced by *L. major* and *L. mexicana*. On Day 28 following start of treatment, *L. major*- (a to c) and *L. mexicana*- (d) infected animals treated by tattooing with PBS vehicle (*left panel*) or OlPC-liposomes (*right panel*) were sacrificed and the skin around the lesion was taken and processed for histology using H&E straining. Numbered areas delineate examples of major findings: 1-epidermal hyperplasia, 2-focal ulceration, 3-dermal fibrosis, 4-inflammation. Arrows point representative examples of amastigote-containing histiocytes. Data show representative pictures of 2 independent experiments.

Histopathological investigations were performed in tattooed mice to better appreciate treatment effect at the cellular and tissue levels. Skin lesions of Day 28 *L. major*- and *L. mexicana*- infected animals (tattooed with PBS vehicle or OlPC-liposomes) were processed for histopathology analysis and representative pictures are presented on Figure 5. *L. major*-infected PBS-tattooed skin sections (5a to 5c, *left panels*) showed moderate focal epidermal hyperplasia, focal ulceration with serocellular exudation (5a, *area 1* and *2*, also shown 400× on 5b *upper panel*) and dermal fibrosis (5a, *area 3*). Extensive and severe dermal mixed inflammation extending in the deep dermis was also seen (5a, *area 4*, also shown 400 and 1000× on 5b and 5c, respectively). The inflammation region was mainly composed of histiocytes (tissue-macrophages) containing a myriad of round intracellular amastigotes measuring 2–3 μm in diameter (examples are pointed with arrows on 5c). Similarly, *L. mexicana*-infected PBS-tattooed (5d left panel) skin sections presented extended area of severe inflammation composed of large histiocytes with few granulocytes and rare lymphocytes (not shown). The histiocytes had marked dilated cytoplasm filled with amastigotes (examples are pointed with arrows).

In contrast, *L. major*-infected OlPC-tattooed skin sections (Figure 5a to 5c, *right panels*) presented focal epidermal hyperplasia and dermal fibrosis (5a, *areas 1* and *3*, respectively), but accompanied with a mild and multifocal dermal inflammation, mainly lymphohistocytic, and mostly free of amastigotes. Likewise *L. mexicana*-infected OlPC-tattooed (5d, *right panel*) skin sections showed mild dermal inflammation composed of histiocytes, granulocytes and rare lymphocytes. Scarce histiocytes containing amastigotes were detectable in the upper dermis.

Despite some remaining parasitized-histiocytes, these histological observations are supportive of an overall excellent efficacy of the tattoo-administered drug at killing intracellular dermal *Leishmania* parasites and reducing inflammation at the lesion site.

## Discussion

This study demonstrates that the use of a tattoo instrument for drug delivery is possible in the treatment of cutaneous leishmaniasis, and that this method can successfully eliminate intracellular parasites at the site of infection. After showing that the selected drug oleylphosphocholine (OlPC) formulated as liposomes could efficiently reach intracellular parasites when in contact with infected macrophages, the activity of the drug was compared *in vivo* in mouse models of Old (*L. major*) and New World (*L. mexicana*) leishmaniasis. Three routes of administrations of the same drug formulation were investigated: systemic (IP) administration, topical administration as a drop, and administration via the tattoo instrument. Evaluation parameters included clinical (lesion sizes) and parasitological parameters (burdens) using quantitative and qualitative methods. In all experiments, the tattooing delivery procedure was the most efficacious at both the clinical and parasitological levels.

Our data support the idea that the mode of administration of a drug can impact its efficacy, suggesting that drugs already approved for the treatment of cutaneous leishmaniasis could be repositioned to be delivered with a tattoo instrument, owing a proper formulation. In addition, previous studies on tattoo-mediated DNA-vaccination have proposed that the minor mechanical injuries caused by the tattoo procedure (hemorrhage, necrosis, inflammation followed by regeneration) could enhance immune response at the humoral and cellular level^6^. The possibility that a non-specific stimulation of the immune system by the tattooing instrument could also contribute to CL lesion healing is interesting and deserves further investigation.

In this study, a liposomal formulation was selected since these particles are prone to ingestion by phagocytic cells such as macrophages, hence increasing treatment efficacy. In experimental models of CL, the therapeutic performance of drug-containing particles targeting phagocytes was shown to greatly depend on their capacity to reach the site of infection intact, particularly following non-parenteral administration^7^. Topical administration of those particles is rendered difficult by the primary barrier of the stratum corneum or by ulcerating skin, which hampers their proper penetration. For example, although a topically administered liposomal formulation of paromoycin has been suggested to improve drug penetration in a pig skin model, its absolute permeation remained relatively low^2^. Similarly in our study, the administration of OlPC-liposomes as a drop on top of the lesions led to partial clinical efficacy which did not translate into parasitological efficacy, possibly due to low skin penetration. As skin surface continuously evolves during the clinical course of CL, the tattooing procedure may represent an elegant solution to transport drug-containing particles across this primary barrier and directly into the infected dermis at all stages of the disease.

Another clear advantage of the tattooing delivery-system is the low amount of drug needed for treatment. We calculated that the total dose needed for tattoo-treatment was ~6–16 times lower than the total dose administered IP. This could translate into a major clinical advantage for patient management, as cost-effectiveness is a criteria closely monitored when it comes to the treatment of neglected diseases like leishmaniasis^8^.

The present study being essentially a proof of concept, a single drug concentration (18 mg/ml) and fixed treatment duration (2 × 5 days) were used for every experiment. The major limitation of this work is the lack of precise knowledge of the dose which is effectively delivered to the dermis during each tattooing session, and the actual fate of the drug following its tattooing. Therefore, additional preclinical PK/PD research is needed in animal models to refine the treatment regimen and best adapt it for clinical use in CL patients. In order to offer a competitive advantage over traditional intra-lesional injections of antimonials, the treatment regimen by tattooing would need to consist of a maximum of one to three sessions. The long half-life and amphiphilic physicochemical characteristics of OlPC make it a potentially good drug candidate to meet this ideal target profile. In addition, the tattoo-mediated treatment of CL could provide a new solution for patients having lesions currently not recommended for treatment (including those caused by *L. major* or those smaller than 5 cm in diameter), and for the management of lesions located in delicate topographies like on the lips, eye lids or ears. This is realistically possible given the excellent efficacy obtained in this study, and because adapted needles for delicate regions already exist and are used in cosmetology for the application of permanent eyeliner and lip contour.

The idea of therapeutic tattooing is not a new concept as “Ötzi the Iceman”, dated c. 3300 BC, was found to bear 57 separate tattoos hypothesized to be of “*therapeutic purpose*”. However, this report is the first making use of tattooing as a mean to deliver a drug product. If proven to be useful in case management of human CL, this technique could lead to new therapeutic interventions in the treatment of other conditions such as psoriasis, skin cancer or other infections.

## Methods

### *In vitro* assays, parasites and infections

#### (i)Assays on promastigotes

Promastigotes of *Leishmania major* LV39 (MRHO/Sv/59/P), *Leishmania mexicana* (MNYC/BZ/62/M379), *Leishmania donovani infantum* LV9 (MHOM/ET/67/HU3), *Leishmania donovani donovani sudanese* 1S2D (MHOM/SD/62/1S-CL2D), *Leishmania donovani archibaldi* 2211 (MHOM/SD/91/D1783; LEM 2211), *Leishmania mexicana amazonensis* LV78 (MPRP/BR/72/M1845), *Leishmania (Viannia) panamensis* (MHOM/87/CO/UA140), *Leishmania (Viannia) guyanensis* (MHOM/SR/87/TRUUS4) and *Leishmania tarantolae* (TarIIWT) in log phase of growth (5 days of culture) were exposed to OlPC in duplicate alongside non-treated controls. Stock solution of OlPC (100 mM) was prepared in endotoxin-free PBS (Wisent) and consecutively diluted (2 × serial dilutions; 125 ul/well in 96 well plates) to obtain final concentrations ranging from 0.1 μM to 51.2 μM in presence of 125 μl of Leishmania culture. Plates were incubated at 25°C for 7 days (stationary phase) in presence of SDM medium pH 7.3 supplemented with 10% heat inactivated FBS and hemine, then Leishmania concentrations were determined by optical density (600 nm)^9^. Parasite concentrations in treated cultures were expressed as percentages of concentration in non-treated cultures.

#### (ii)Cell culture

Murine macrophages cells line B10R^10^ and J774A.1 (ATCC) were maintained in culture in DMEM (B10R) or RPMI (J774A.1) supplemented with final concentration of 10% decomplemented fetal bovine serum (FBS - Gibco) and 1× penicillin/streptomycin/glutamine (PSG - Wisent). Cells were passed every two days with starting concentration for B10R: 0.3 × 10^6^/8 ml/100 mm^2^ dish (BD falcon); and J774A.1: 0.7 × 10^6^/8 ml/100 mm^2^ dish (BD falcon). Cell detachment was done using rubber cell scrapper (B10R) or re-suspended with culture medium (J774A.1).

### Determination of % of infected cells by light microscopy

One coverslip (Fisher) was added/well in 24 wells plate (BD Falcon). B10R (0.1 × 10^6^ cells/1 ml of DMEM 10% FBS/well in 24 wells plate) and J774A.1 (0.2 or 0.1 × 10^6^ cells/1 ml of RPMI 5% FBS/well in 12 wells plate) were plated on the day before the experiment. The next day medium was replaced by 300 μl of fresh medium containing *Leishmania major (NIH S (MHOM/SN/74/Seidman) clone A2)* or *L. mexicana* *(MNYC/BZ/62/M379)* in 1:20 (B10R) or 1:10 (J774A.1) ratios, assuming that cells had divided once since plating (i.e. considering double amount of cells compared to the starting numbers). After 6 h of infection, cells were washed with PBS and coverslips were put in presence of OlPC-liposome concentrations. Cells were incubated for an additional 18 hrs. After 24 h of culture, cells were washed twice with PBS and coverslips were collected and stained with Diff Quick (Behring #84132-1A). Subsequently dried coverslip were mounted on slides with Permount media (Fisher) and % of infected cells were determined using 3–5 count of 100 cells by light microscopy (Leica).

### Animals

Balb/c female mice of 6 to 9 weeks of age (weights of about 20 g) were purchased from Charles River (Montreal, Canada) and housed at McGill University. All experiments were performed in accordance to the guidelines of the Canadian Council on Animal Care. Animal protocols were approved by the Animal Care committee of McGill University (UACC); protocol number: AUP#6088.

### *In vivo* infections

Mice (10 mice per/group) were shaved at tail base and injected subcutaneously (s.c.) with 50 μl of sterile saline containing 10 × 10^6^ of *Leishmania major* Friedlin-luciferase (stably transfected with luciferase reporter gene)^11^ or with *Leishmania mexicana* using a 1 ml syringe and 27 G^1/2^ needle. Mice were then checked regularly to monitor the appearance and growth of the lesions until they reached ~5 mm^2^ in surface (average 8–12 weeks post-infection). Following lesion development, mice were selected according to lesion size to ensure similar levels of infection and were divided into groups (6–9 mice showing lesion/group) for treatment. Control groups were treated with PBS while treatment groups were treated with OlPC-liposomes. Each group was treated daily for 10 days (2 × 5 days) with the permanent make-up (PMU) delivery system, by intraperitoneal injection, or by simply dropping the liposomal drug on the lesion (see below).

### Substance and Substance delivery

Oleylphosphocholine (OlPC; Dafra Pharma Research and Development, Belgium) was formulated in liposome and stored as previously described^4^. The OlPC -liposomes were used directly from the stock (18 mg/ml, final concentration) for tattooing or freshly diluted in medium for the *in vitro* experiments.

Tattooing-mediated drug delivery was performed using a commercial professional permanent make-up apparatus (Nouveau contour Digital 1000 machine, Micro-Pigmentation Inc., Missisauga, Canada) and needles (Nouveau contour Needle #5 Magnum (5-needles head)). The substances (PBS vehicle or anti-leishmanial therapy) were administered on anesthetized mice (isoflurane mask) to facilitate the procedure. Each tattooing session was done by performing 12 times repeated two second lasting administrations with the 5 needles oscillating at 100 Hz (i.e. 100 punctures per second), for a total of (5 × 12 × 2 × 100) 12000 punctures; excess drug was not wiped off after the procedure. We estimate that ~2–5 μl of OlPC-liposomes (36–90 μg) is injected into the skin during every tattooing session^3^. The device was adjusted to allow exposure of 1.5 mm of the needle tip beyond the barrel guide. Gentle pressure was used to facilitate penetration of the needle into the skin resulting in the deposition of the substances into the dermis^5^. Topical treatments were done by applying 50 μl of liposomal drug (i.e. 900 μg of OlPC) on the lesion for 30 seconds on anesthetized mice (isoflurane mask) before waking up the animal. Intraperitoneal treatments were performed at the dose of 30 mg/kg for 10 days (2 × 5 days) without anesthesia. Following each drug administration mice were closely monitored for evidence of inflammation, pain and distress. Animal weights were also measured as signs of toxicity.

### *In vivo* treatment evaluation

Treatment efficacy was evaluated using three different parameters: (i) lesion areas; (ii) skin histolopathology; and (iii) tissue parasite burden as measured by luciferase activity and dilution assays.

#### (i) Lesion areas

For each experiment pictures of the lesions were taken on Day 0, 7, 14, 21 and 28 to document the evolution of the sizes. Lesion areas were individually calculated from the pictures using the Adobe photoshop® software and were expressed in mm^2^. Specifically, the ruler tool was first used to determine the pixel number in 1 cm on the ruler present on a given picture. These values were used to set the measurement scale (Pixel number = L1 valor, Logical length = 1, Logical unit = cm). Subsequently, the lesion borders were delimited using the lasso tool and the measurement was recorded. A mouse with no apparent lesion was given a lesion area of the smallest area possible to draw ~ 0.0007 ± 0.00042 mm^2^.

#### (ii) Histopathology

After Day 28, the infected animals were humanely euthanized in a CO_2_ chamber and the skin around the lesion was taken and washed in PBS, fixed in 10% neutral formalin, processed and embedded in paraffin. Blocks were cut at 4 μm, mounted on slides and stained with H&E and Giemsa.

#### (iii) Quantification of tissue parasite burdens

Limiting dilution assay was done as previously described^12^,^13^, with some modifications. Briefly, on Day 28 mice were euthanized and individual lesions were disinfected, excised and homogenized in 1 ml of sterile PBS. Tubes were spun down (500 RPM for 30 seconds) and 100 μl of the lysate was diluted again in 10 ml of PBS (1:100). A hundred μl of extracted parasites (in 1 ml or 100 times diluted) were serially diluted in a 96 well plate in duplicate and put in culture. Eight days later the number of viable parasites was determined from the highest countable dilution using an inverted microscopy. This highest dilution was multiplied by a factor 10 (1000 μl/100 μl) or 1000 (1000 μl/100 μl × 100) to obtain the parasite burden. Lesions infected with luciferase-labeled parasites were processed as above, then homogenized material from each lesion was spun down (13,000 rpm/10 min) and the pellet were frozen at −80°C. Frozen pellets were lysed with 30 μl of Cell Culture Lysis Reagent (Promega) diluted 1:5 in ddH_2_O, vortexed, incubated on ice 15 min and spun down at 13000 rpm/10 min at 4°C. Five μl of lysate were then mixed with 90 μl of Luciferase Assay Reagent (Promega) and luciferase activity were determined using a Mini Lumat LB 9506 luminometer (EG&G, Berthold, Germany). Protein concentrations were measured using Bradford reagent (Bio-Rad). Results were reported as relative light units (RLU)/μg of protein.

### Statistical analysis

IC50 on promastigotes were calculated by non-linear regression using GraphPad Prism5® software. For the statistical analysis in biological assays, means ± standard errors of the mean (SEM) were calculated by the GraphPad Prism5® software. Multiple comparisons between groups were made with a one-way analysis of variance (ANOVA) followed by Dunnet and Tukey tests. P values of less than 0.05 were considered significant (*).

## Author Contributions

Project conception and design (A.F., S.S., M.O.); Acquisition, analysis, and interpretation of data (M.T.S., M.P., C.M., M.O., A.F.); Figures and tables preparation (M.T.S., M.P., T.B., A.F.); writing of main manuscript (M.T.S., A.F.); critical revision of manuscript (M.P., S.S., T.B., M.O.); All authors approved the manuscript prior to submission.

## Supplementary Material

Supplementary Information Supplementary Figure 1

## Figures and Tables

**Table 1:** *In vitro* anti-leishmanial activity of OlPC against *Leishmania* spp. promastigotes

*Leishmania* spp. and strain	IC50 (95% CI), μM
Old World strains	
*L. donovani archibaldi 2211 (MHOM/SD/91/D1783; LEM 2211)*	8.0 (6.2–10.5)
*L. donovani donovani sudanese 1S2D (MHOM/SD/62/1S-CL2D)*	4.0 (2.7–5.7)
*L. donovani infantum LV9 (MHOM/ET/67/HU3)*	3.0 (1.6–6.4)*
*L. major LV39 (MRHO/Sv/59/P)*	1.1 (0.9–1.3)
New World strains	
*L. mexicana amazonensis LV78 (MPRP/BR/72/M1845)*	5.7 (3.2–12.8)*
*L. guyanensis (MHOM/SR/87/TRUUS4)*	3.0 (2.3–3.9)
*L. mexicana (MNYC/BZ/62/M379)*	2.1 (1.3–3.7)
*L. panamensis (MHOM/87/CO/UA140)*	2.7 (2.2–3.4)
*L. tarantolae (TarIIWT)*	7.8 (6.6–9.2)

*Confidence intervals were conservatively defined by concentrations resulting in ≤5% and ≥95% reduction in parasite density, as they could not be reliably calculated by the software. Data shown are the mean of the three independent experiments made in duplicates.

## References

[b1] WHO technical report series; no. 949. Control of the leishmaniases. Report of a meeting of the WHO Expert Committee on the Control of Leishmaniases, Geneva, 22–26 March 2010; © World Health Organization, 2010; WHO Press, Switzerland. http://whqlibdoc.who.int/trs/WHO_TRS_949_eng.pdf (accessed on 23/05/2012).

[b2] Carneiro G., Aguiar M. G., Fernandes A. P. & Ferreira L. A. Drug delivery systems for the topical treatment of cutaneous leishmaniasis. Expert. Opin. Drug Deliv. 9, 1083–1097 (2012). 22724539 10.1517/17425247.2012.701204

[b3] Bins A. D. *et al.* A rapid and potent DNA vaccination strategy defined by in vivo monitoring of antigen expression. Nat. Med. 11, 899–904 (2005). 15965482 10.1038/nm1264

[b4] Fortin A. *et al.* Efficacy and tolerability of oleylphosphocholine (OlPC) in a laboratory model of visceral leishmaniasis. J. Antimicrob. Chemother. 67, 2707–2712 (2012). 22782488 10.1093/jac/dks273

[b5] Gopee N. V. *et al.* Response of mouse skin to tattooing: use of SKH-1 mice as a surrogate model for human tattooing. Toxicol. Appl. Pharmacol. 209, 145–158 (2005). 15913690 10.1016/j.taap.2005.04.003

[b6] Pokorna D., Rubio I. & Muller M. DNA-vaccination via tattooing induces stronger humoral and cellular immune responses than intramuscular delivery supported by molecular adjuvants. Genet. Vaccines. Ther. 6, 4 (2008). 18257910 10.1186/1479-0556-6-4 PMC2267179

[b7] Romero E. L. & Morilla M. J. Drug delivery systems against leishmaniasis? Still an open question. Expert. Opin. Drug Deliv. 5, 805–823 (2008). 18590464 10.1517/17425247.5.7.805

[b8] Vanlerberghe V. *et al.* Drug policy for visceral leishmaniasis: a cost-effectiveness analysis. Trop. Med. Int. Health 12, 274–283 (2007). 17300636 10.1111/j.1365-3156.2006.01782.x

[b9] St George S. *et al.* Novel compounds active against *Leishmania major*. Antimicrob. Agents Chemother. 50, 474–479 (2006). 16436699 10.1128/AAC.50.2.474-479.2006 PMC1366913

[b10] Radzioch D. *et al.* Genetic resistance/susceptibility to mycobacteria: phenotypic expression in bone marrow derived macrophage lines. J. Leukoc. Biol. 50, 263–272 (1991). 1856597 10.1002/jlb.50.3.263

[b11] Roy G. *et al.* Episomal and stable expression of the luciferase reporter gene for quantifying Leishmania spp. infections in macrophages and in animal models. Mol. Biochem. Parasitol. 110, 195–206 (2000). 11071276 10.1016/s0166-6851(00)00270-x

[b12] Lima H. C., Bleyenberg J. A. & Titus R. G. A simple method for quantifying Leishmania in tissues of infected animals. Parasitol. Today 13, 80–82 (1997). 15275128 10.1016/s0169-4758(96)40010-2

[b13] Gomez M. A. *et al.* Leishmania GP63 alters host signaling through cleavage-activated protein tyrosine phosphatases. Sci. Signal. 2, ra58 (2009). 19797268 10.1126/scisignal.2000213

