# Supplementary material for: Drug Delivery by Tattooing to Treat Cutaneous Leishmaniasis
Source: Sci Rep. 2014 Feb 24;4:4156. doi: 10.1038/srep04156 (PMC3932479; doi:10.1038/srep04156)
Supplement: Supplementary Information — Supplementary Figure 1 [file srep04156-s1.pdf]

Supplementary Information

DRUG DELIVERY BY TATTOOING TO TREAT CUTANEOUS LEISHMANIASIS

Marina Temi Shio, Marilene Paquet, Caroline Martel, Tom Bosschaerts, Stef Stienstra, Martin Olivier and Anny Fortin*

*Corresponding author: anny.fortin@mcgill.ca

Figure S1

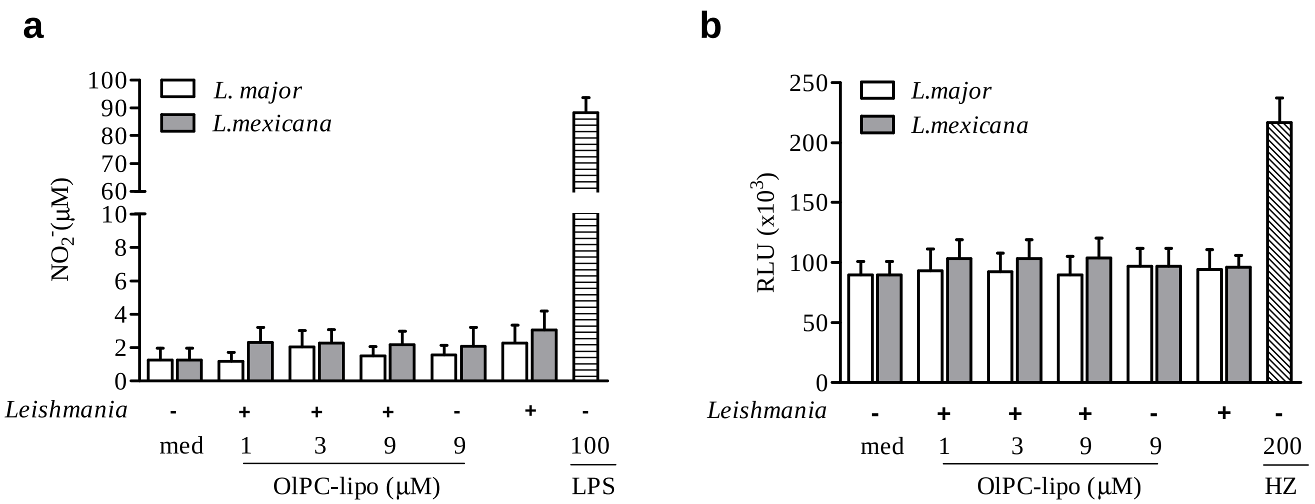

Supplementary Figure 1. OlPC does not induce NO or ROS production in *Leishmania*-infected macrophage-derived cell lines. *L. mexicana* or *L. major*–infected B10R cells were treated with medium (med) or with indicated concentrations of OlPC liposomes (OlPC-lipo) alongside with 100 ng/ml LPS or 200 μg/ml hemozoin (HZ) as positive controls. After 24 h the supernatants were collected to determine the concentrations of (**a**) nitrite by Griess assay or (**b**) ROS by exposure to a cell permeable DCFH-DA for different times for 3 hrs. Data are expressed as mean + SEM, n=2 - 4 experiments.
